# Advances in understanding red blood cell modifications by *Babesia*

**DOI:** 10.1371/journal.ppat.1010770

**Published:** 2022-09-15

**Authors:** Hassan Hakimi, Junya Yamagishi, Shin-ichiro Kawazu, Masahito Asada

**Affiliations:** 1 National Research Center for Protozoan Diseases, Obihiro University of Agriculture and Veterinary Medicine, Obihiro, Hokkaido, Japan; 2 Department of Veterinary Pathobiology, College of Veterinary Medicine, Texas A&M University, College Station, Texas, United States of America; 3 Division of Collaboration and Education, International Institute for Zoonosis Control, Hokkaido University, Sapporo, Japan; Joan and Sanford I Weill Medical College of Cornell University, UNITED STATES

## Abstract

*Babesia* are tick-borne protozoan parasites that can infect livestock, pets, wildlife animals, and humans. In the mammalian host, they invade and multiply within red blood cells (RBCs). To support their development as obligate intracellular parasites, *Babesia* export numerous proteins to modify the RBC during invasion and development. Such exported proteins are likely important for parasite survival and pathogenicity and thus represent candidate drug or vaccine targets. The availability of complete genome sequences and the establishment of transfection systems for several *Babesia* species have aided the identification and functional characterization of exported proteins. Here, we review exported *Babesia* proteins; discuss their functions in the context of immune evasion, cytoadhesion, and nutrient uptake; and highlight possible future topics for research and application in this field.

## Introduction

### *Babesia* parasites modify the host red blood cell

*Babesia* species are protozoan parasites belonging to the phylum Apicomplexa and are transmitted by ticks. More than 100 *Babesia* species have been reported, which infect mammalian and avian hosts, and several species are known to parasitize domestic animals such as cattle, horses, sheep, goats, as well as dogs [1]. Bovine *Babesia* species (*B*. *bovis*, *B*. *bigemina*, *B*. *divergens*, *B*. *major*, and *B*. *ovata*) capable of mediating disease are widely distributed in temperate and tropical regions in the world. It is estimated that 1.2 billion cattle are at risk of infection, and bovine babesiosis is a major cause of economic loss within the beef and dairy industries, thus highlighting the significance of this protozoan parasite in veterinary medicine [2]. Several *Babesia* species, including *Babesia microti* and *B*. *divergens*, have gained attention as pathogenic species for emerging zoonotic diseases [3].

*Babesia* sporozoite stage parasites are released from the salivary glands of infected vector ticks during a blood meal and directly invade red blood cells (RBCs) to become pear-shaped “pyriform” intraerythrocytic stage parasites [4,5,6]. The pyriform parasites rapidly replicate by binary fission, leading to paired parasites inside the RBC. Some clades of *Babesia*, such as *B*. *microti* and *B*. *duncani*, form 4 merozoites per division, which result in the appearance of the Maltese cross form [7]. This multiplication and the following destruction of the host RBC during parasite egress lead to pathologies including fever, anemia, jaundice, and hemoglobinuria. Although most apicomplexan parasites infect nucleated host cells, *Babesia* and a few closely related genera such as the malaria parasite *Plasmodium* have evolved to parasitize enucleated RBCs. During invasion and subsequent development, the parasite modifies host RBCs by exporting proteins. Modification of nucleated host cells is known for many apicomplexan parasites, and exported proteins manipulate host cell processes such as transcription and kinase activity [8]. *Babesia* and *Plasmodium* reside inside anucleate RBCs (*Plasmodium* also has a liver stage, but *Babesia* solely multiply inside RBCs), and, therefore, the role of exported proteins is likely different from apicomplexans, which infect nucleated cells. The repertoire of exported proteins is called the “exportome”, and in the instance of *Plasmodium*, hundreds of such proteins have been identified within numerous expanded gene families [9,10]. Extensive studies have described the role of *Plasmodium* exportome proteins in reinforcing the RBC cytoskeleton, altering the RBC surface to evade immune recognition, inducing cell adhesive properties, and changing the RBC membrane permeability to allow the uptake of nutrients and release of parasite metabolic waste products [11]. Since the exportome is important for the parasite survival and its pathogenicity, these proteins are considered to be drug or vaccine targets [11].

Despite the similarities to *Plasmodium* infection, less is known regarding the *Babesia* exportome, and the degree of overlap, if any, in the repertoire of exported proteins. While it is known that infected RBCs (iRBCs) are modified by *Babesia* parasites, such as to induce RBC adhesive properties and alter permeability, prior to the past few years, only a few exported proteins were identified and functional analysis was scarcely done [12,13]. However, the recent completion of genome sequences and the establishment of transfection systems for several *Babesia* species has aided characterization of the exportome, and the functions of several exported proteins have been partially revealed.

### *Babesia* exportome and the motifs responsible for protein export

**Fig 1:**
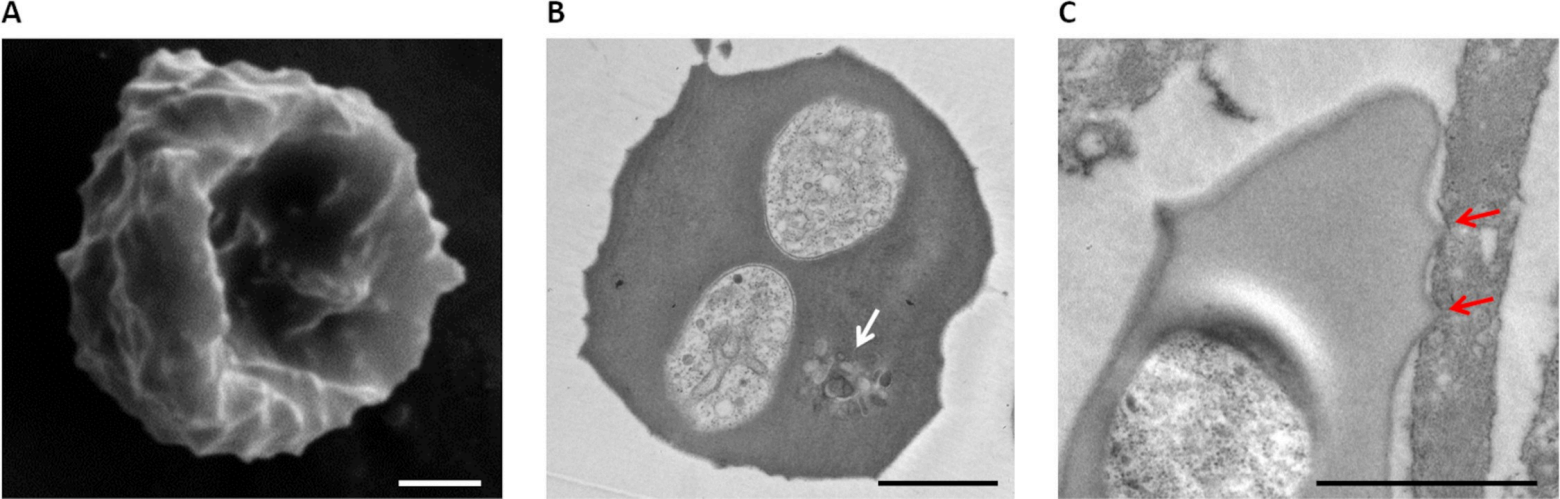
Electron microscopy images of *B*. *bovis*-infected RBCs. Scanning electron microscopy (A) and TEM (B) and (C) of *B*. *bovis*-iRBC binding to bovine brain endothelial cells. Membranous structures are seen in the TEM image and indicated by a white arrow. The red arrows show the ridges mediating binding of iRBCs to endothelial cells. Scale bar = 1 μm. iRBC, infected RBC; RBC, red blood cell; TEM, transmission electron microscopy.

Before the identification of the exportome in *Babesia* parasites, modification of iRBC by the parasite was known from studies on cerebral babesiosis, a fatal complication in cattle infected with the most virulent bovine *Babesia* species, *B*. *bovis* [14]. Sequestered iRBCs were found in the cerebral microcapillary of infected cattle, mediated by the cytoadhesion of iRBCs to brain microcapillary endothelial cells. *Plasmodium falciparum*, a causative agent of human malaria, produces similar clinical symptoms, called cerebral malaria [15]. *B*. *bovis* and *P*. *falciparum* iRBCs both show protrusions on the surface of the RBC, which, based upon their topology, are called ridges in *B*. *bovis* iRBCs (Fig 1A) and knobs in *P*. *falciparum* iRBCs [16,17]. During and following invasion, *Plasmodium* proteins are secreted from rhoptries and dense granules into the parasitophorous vacuole and are trafficked to the iRBC cytoplasm and surface [11]. Several proteins responsible for knob formation have been identified [11]. Many exported proteins are regulated by a host targeting signal “PEXEL-motif”, which consists 5 amino acids RxLx(x)E/Q/D (x represents any amino acids) located downstream of a signal peptide that mediates translocation to the endoplasmic reticulum (ER) [18,19]. Within the ER, PEXEL-containing proteins are proteolytically cleaved at the PEXEL motif and the new N-terminus is recognized for targeting to the parasitophorous vacuole and translocation across the parasitophorous vacuole membrane (PVM) by a *Plasmodium*-specific translocon [20,21]. Some of the proteins in the RBC compartment are transported to vesicle like structures of parasite origin within the RBC cytoplasm, termed Maurer’s clefts in *P*. *falciparum*, for either final residence or subsequent delivery to a functional destination within the RBC [10]. In addition, *P*. *falciparum* exports several PEXEL-negative proteins (PNEPs) [22]. PNEPs typically contain a transmembrane domain or N-terminal signal sequence, and these proteins are trafficked either in a soluble or membrane-bound form [23,24].

Unlike *Plasmodium* parasites, *Babesia* and its close relative *Theileria* break down their PVM within minutes after completion of invasion [25–27]. The lack of a PVM precludes the need for a translocon for the export of proteins across this membrane. Maurer’s cleft-like structure have not been identified in *Babesia* parasites; however, membranous structures have been observed in electron microscopy images and are proposed to be either vesicles responsible for protein trafficking to the RBC or the remains of the dissociated PVM ([25,27]; Fig 1B). Due to the structural differences of RBCs modified by *Babesia* versus *Plasmodium*, it is plausible that the export machinery is simpler for *Babesia* and that motifs mediated trafficking differ between the 2 parasite genera.

**Fig 2:**
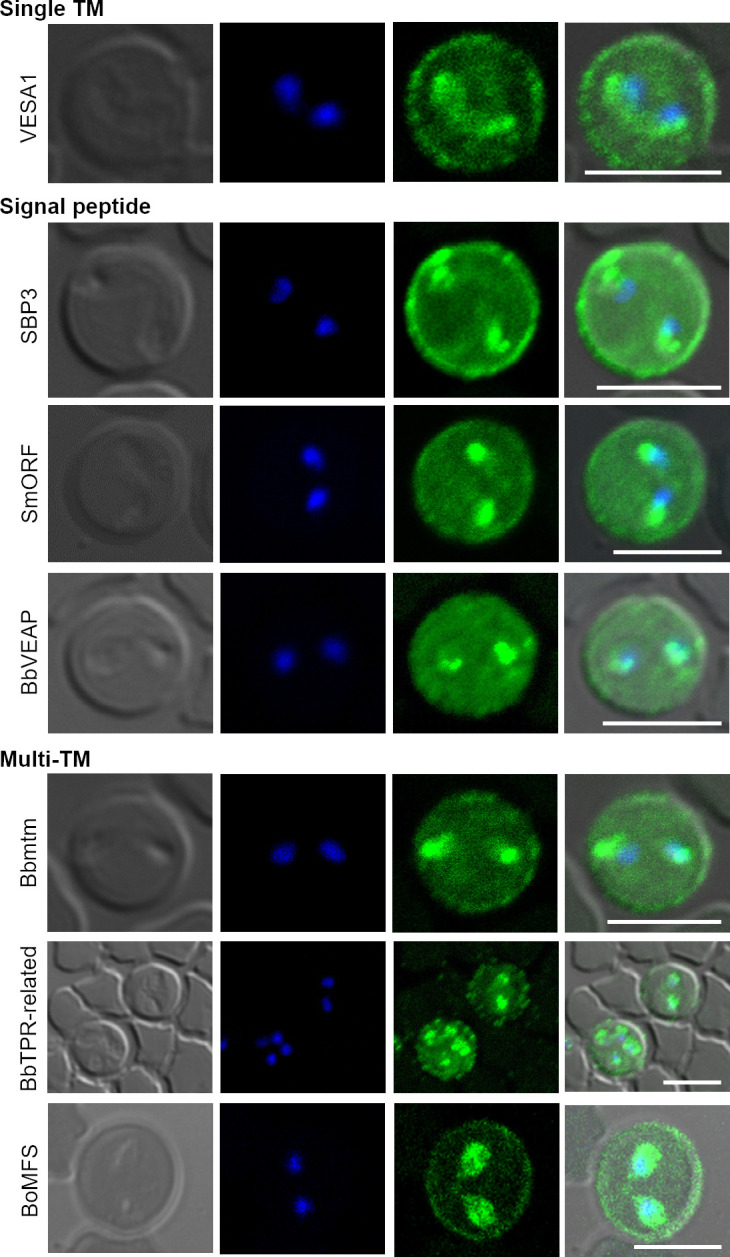
Immunofluorescence microscopy of transgenic parasites expressing myc-tagged exported protein. Myc-tagged proteins were episomally expressed in *B*. *bovis* (VESA1, SBP3, SmORF, BbVEAP, Bbmtm, and BbTPR-related) or *B*. *ovata* (BoMFS). The expression plasmid was constructed and transfected by electroporation as described [32,33], and transgenic parasites were selected using WR99210. Thin smears of cultured parasites were prepared for indirect immunofluorescence microscopy, fixed with a 1:1 acetone:methanol mixture, and reacted with anti-myc mouse monoclonal antibody (9B11, Cell Signaling) followed by Alexa Fluor 488-conjugated goat anti-mouse IgG (Invitrogen, green). Nuclei were stained with Hoechst 33342 (Hoechst, blue). Scale bar = 5 μm. BbVEAP, *B*. *bovis* VESA1-export associated protein; IgG, immunoglobulin G; SBP3, spherical body protein 3; SmORF, Small Open Reading Frame; VESA1, variant erythrocyte surface antigen 1.

The first identified *Babesia* exportome protein was the *B*. *bovis* variant erythrocyte surface antigen 1 (VESA1) ([28]; Fig 2). It was known that antibodies raised against *B*. *bovis* iRBC can mediate their agglutination, leading to speculation of parasite-encoded antigens on the RBC surface [29]. A family of highly antigenic variable proteins were later identified on the iRBC surface and named VESA1 [30]. Currently, it is known that VESA1 is the product of a multigene family and localizes on the ridges of the iRBCs, discussed further below [31].

Another *Babesia*-exported protein identified from early studies was an immunodominant *B*. *bovis* protein, spherical body protein 1 (SBP1, originally named Bb-1) [34]. Immunofluorescence assays revealed that SBP1 was detected at the permeabilized iRBC membrane and inside the parasite but not using preparations of unfixed iRBC, indicating that SBP1 was localized at the cytoplasmic side of iRBCs [35]. Immunoelectron microscopy analysis revealed SBP1 within the spherical bodies of the parasites, which are unique organelles for *Babesia* and *Theileria* and appear to be analogous to dense granules of other apicomplexan parasites [36]. Antibodies raised against spherical bodies identified 2 more proteins, SBP2 and SBP3 ([36,37]; Fig 2). Similar to SBP1, SBP2 and SBP3 localized on the cytoplasmic face of iRBCs and spherical bodies of the parasites. Currently, a fourth protein (SBP4) has been deposited in GenBank, and the localization in iRBCs has been confirmed [38,39]. Although SBPs contain a signal peptide and PEXEL-like motif (PLM), they are heterogeneous in structure; thus, future functional characterizations might inspire a less generic renaming of these proteins. While the export of VESA1 and SBPs were shown experimentally, a PEXEL motif was not found in these proteins.

**Fig 3:**
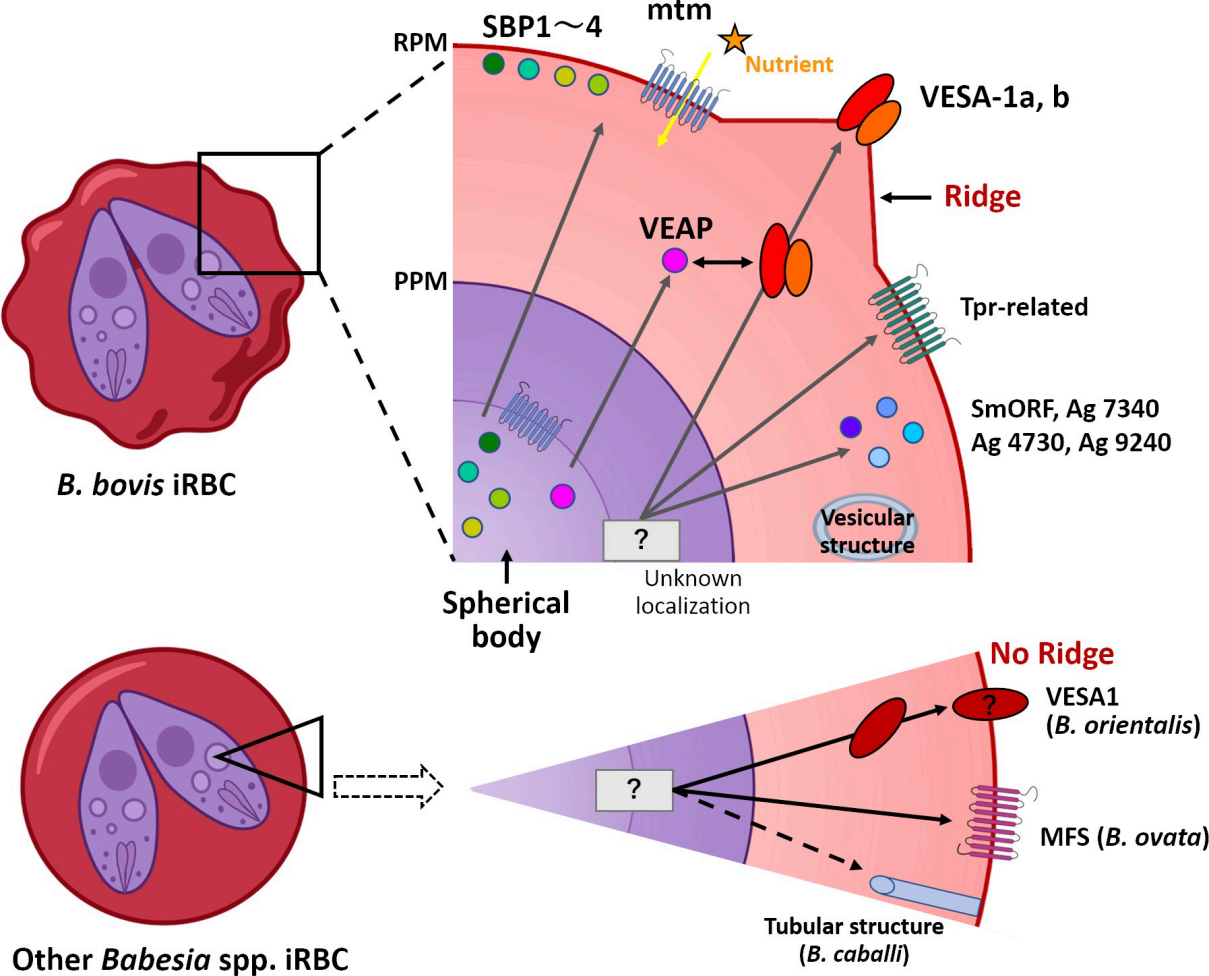
*Babesia* iRBC. *Babesia* parasites export numerous proteins to remodel the host RBC using secretory pathway. Several of these proteins are routed through spherical bodies, while others are either directly exported to RBC or deposited in unknown secretory organelles. *B*. *bovis* produces ridges on the surface of iRBCs to express VESA1 that mediates sequestration and immune evasion. Of spherical body proteins, SBP1, SBP2, SBP3, and SBP4 are associated with the RBC membrane; VEAP is released to the RBC cytoplasm and is essential for parasite growth and VESA1 export; mtm integrates into the RBC membrane and is likely responsible for nutrient uptake. Vesicular structures in the RBC are produced following parasite invasion that might have a role in protein export. Regarding other *Babesia* spp., VESA1 expression in iRBC was shown for *B*. *orientalis* and MFS export to RBC for *B*. *ovata*, though their functions remain to be determined. iRBC, infected red blood cell; mtm, multi-transmembrane protein; PPM, parasite plasma membrane; RPM, RBC plasma membrane; SBP1, spherical body protein 1; SmORF, Small Open Reading Frame protein; VEAP, VESA1-export associated protein; VESA1, variant erythrocyte surface antigen 1.

To date, 13 proteins including 5 protein families have been experimentally confirmed to be exported by *Babesia* (Table 1 and Fig 3). The completion of the genome sequence of *B*. *bovis* enabled bioinformatic approaches to find new exported proteins [40]. For example, Gohil and colleagues selected exportome candidates by virtue of their possessing a signal peptide, transmembrane domain, GPI anchor, and domains/features of extracellular proteins [41]. They found 214 proteins with signal peptides but without transmembrane domains or GPI anchors and concluded with the identification of 3 novel candidate exportome proteins (Gene IDs: BBOV_II007340, BBOV_II004730, and BBOV_IV009240). Another study used a bioinformatic approach to describe a PLM that is consistent with an exportome prediction [42]. The PEXEL-related signal was originally identified from another apicomplexan parasite, *Toxoplasma gondii* and named PLM [43]. This motif consists of Rx(x)L and is cleaved by an aspartyl protease, similar to *Plasmodium* parasites. PLM is required for *Toxoplasma* protein trafficking to dense granules and PVM binding. Pelle and colleagues searched for PLM in *B*. *bovis* proteins and found that a Rx(x)L motif can be found at a higher frequency than permutated control sequences. PLM preceded by a signal peptide can be found in SBPs and Small Open Reading Frame proteins (SmORF) (Fig 2). Using the SmORF or SBP2t11 (a protein coded by one of the truncated copies of SBP2) N- terminal sequences fused to a fluorescence protein marker, they demonstrated that a signal sequence is sufficient to export the fluorescence reporter protein to the iRBC. A mutated PLM resulted in a reduced retention of proteins within the spherical bodies, suggesting that the PLM works as a retention signal in spherical bodies for proper protein maturation and release and thus serves a different function than the PEXEL system in *Plasmodium* [42]. Additionally, these proteins were proteolytically processed at the signal sequence and PLM [42]. When SmORF with a complete PLM was expressed in *Plasmodium*, the protein was proteolytically processed and exported across the PVM to the RBC [42]. These results suggest that even though there is a functional difference in the PLM/PEXEL motif between these parasites, the trafficking pathway of *Babesia* PLM-positive proteins is related to PEXEL-dependent trafficking in *Plasmodium* [42].

Another recent bioinformatic approach used prediction by machine learning [44]. This study used Gohil’s 214 proteins as an export-positive training dataset and predicted an exportome for several *Babesia* species. Using 3 prediction algorithms, they estimated an additional 144 potential exportome proteins for *B*. *bovis*; and 371 and 196 exportome proteins for *B*. *bigemina* and *B*. *canis*, respectively (with probability more than 70%). This study was done in silico, and, therefore, confirmatory cellular studies are necessary. In summary, advances in computational biology have given new opportunity to describe the *Babesia* exportome.

**Table 1:** Experimentally confirmed *Babesia* exportome proteins.

Gene product	Gene ID	Function	Number of TM domain	SignalP	RXL or RXXL motif	RXL or RXXL position	*Babesia* species^#^	Refs.
Variant Erythrocyte Surface Antigen 1 (VESA1)	Multigene family protein	Antigenic variation, Cytoadhesion	1	Null	ARIFLGSV*	281-284	Bb	[31]
Small Open Reading Frame Protein (SmORF)	Multigene family protein	Unknown	0	Signal peptide	LREELPSD**	92-95	Bb	[42]
Multi-transmemebrane protein (mtm)	Multigene family protein	Linked to blasticidin S resistance	10	Null	VRYLMPI***	24-26	Bb	[33]
Major Facilitator Superfamily (MFS)	Multigene family protein	Unknown	9	Null	KRQLTML****	66-68	Bbig, Bd, Bo	[33]
Tpr-related protein	Multigene family protein	Unknown	10	Null	Not detected*****		Bb	[33]
Spherical Body Protein 1 (SBP1)	BBOV_II002880	Unknown	0	Signal peptide	NRRLATI	125-127	Bb	[34,35]
Spherical Body Protein 2 (SBP2)^$^	BBOV_II000740^$^	Unknown	0	Signal peptide^$^	Not detected^$^		Bb	[36]
Spherical Body Protein 3 (SBP3)	BBOV_I004210	Unknown	0	Signal peptide	VRYLINT	134-136	Bb, Bbig, Bd, Bo, Bx	[37]
Spherical Body Protein 4 (SBP4)	BBOV_IV005390	Unknown	0	Signal peptide	HRILKTS	68-70	Bb, Bbig, Bo, Bx	[38,39]
VESA1-export associated protein (BbVEAP)	BBOV_III004280	Essential for parasite growth and cytoadhesion	0	Signal peptide	DRVLLES	185-187	Bb, Bbig, Bd, Bo, Bx	[33]
Putative erythrocyte membrane-associated antigen	BBOV_II007340	Unknown	0	Signal peptide	Not detected		Bb, Bbig, Bd, Bo, Bx, Bm	[41]
DnaJ domain-containing hypothetical protein	BBOV_II004730	Unknown	0	Signal peptide	SRLLCGA	27-29	Bb, Bbig, Bd, Bo, Bx, Bm	[41]
Leucine zipper domain containing hypothetical protein	BBOV_IV009240	Unknown	0	Signal peptide	GRTLTPG	75-77	Bb, Bbig, Bd, Bo, Bx, Bm	[41]

^#^Bb, *B*. *bovis*; Bbig, *B*. *bigemina*; Bd, *B*. *divergens*; Bo, *B*. *ovata*; Bx, *Babesia* sp. Xinjiang; Bm, *B*. *microti*.

*BBOV_IV005680.

**BBOV_III011930.

***BBOV_III000060.

****BOVATA_014390.

*****BBOV_III011910.

^$^One complete copy and 12 truncated copy genes exist. Among the truncated copies of SBP2, SBP2t11 has a signal peptide sequence and PLM and was confirmed to be exported.

### Immune evasion and cytoadhesion

To establish an infection in the host, *Babesia* parasites must evade immune recognition, including avoidance of an antibody-mediated immune response or direct clearance of iRBCs in the spleen. The spleen plays a critical role in recognizing and clearing abnormal and aged RBCs, as well as RBCs altered due to infection. In the case of *B*. *bovis*, avoidance of splenic clearance is achieved by the display of parasite-encoded ligands on the surface of the iRBCs, which confer capillary endothelial adhesion and sequestration in internal organs [45]. However, in this manner, the parasite exposes itself to a possible antibody-mediated immune response, which it in turn evades over the course of an infection by sequentially switching the expression of the surface ligands with other members of a repertoire of antigenically variant surface proteins. Antigenic variation in *Babesia* parasites was first described in the rodent parasite *B*. *rodhaini* [46], and following in *B*. *bigemina* [47], and was suggested to occur in *B*. *bovis* by the parasite-encoded proteins on the surface of iRBC that were named VESA1 [31,48,49]. VESA1 is a heterodimeric protein encoded by the multicopy *ves1α* and *ves1β* gene families in *B*. *bovis* [31,50]. The first genome sequence of *B*. *bovis* revealed 119 copies of *ves1* genes [40], which, with improvement of genome assembly, increased to 133 genes consisting of 81 *ves1α*, 48 *ves1β*, and 4 unclassified *ves1* [51]. In the *B*. *bovis* genome, *ves1α* and *ves1β* gene pairs are located in a divergent orientation and are expressed at a site called locus of active transcription (LAT) [52]. It is believed that *ves1* genes show monoallelic expression [53] in which an active LAT containing a *ves1α* and *ves1β* pair are simultaneously expressed by a bidirectional promoter located within the LAT intergenic region [54]. Antigenic variation and the resulting immune evasion in *B*. *bovis* occur through epigenetic in situ switching of transcribed *ves1* and genomic recombination, which results in mosaicism of *ves1* genes [55].

Sequestration of *B*. *bovis* is mediated by interaction of iRBC surface ridges with bovine endothelial cells (Fig 1C) [16,56,57]. The sequestered iRBCs can disrupt blood flow in internal organs and in the brain, which causes cerebral babesiosis [16]. It was proposed that VESA1 is responsible for cytoadhesion of iRBCs based upon the observations that VESA1 proteins are clustered on ridges, and specific monoclonal antibodies against VESA1 inhibited binding of iRBCs to endothelial cells and reversed binding of cytoadhered iRBCs [57]. Additionally, chemical disruption of VESA1 export or trypsin treatment to remove surface proteins resulted in inhibition of binding, which was regained when VESA1 was repopulated on the RBC [56]. Further transcriptomics analysis supported a VESA1 role in pathogenesis [58]. VESA1 has a single transmembrane domain, a large extracellular domain, and a short cytoplasmic tail [31,50], a structure that superficially resembles the *P*. *falciparum* RBC surface adhesin, erythrocyte membrane protein 1 (PfEMP1). The extracellular region of VESA1 proteins possess a cysteine- and lysine-rich domain and variant domain conserved sequences [31,59]. While the role of PfEMP1, the specific binding domains, and the host receptors are well documented for cerebral and pregnancy-associated malaria [60–62], the role of binding domains, receptors, and the impact of VESA1 for sequestration remain to be determined for *Babesia*.

Two additional exported proteins were shown to be involved in cytoadhesion of *B*. *bovis* iRBC, SBP2t11, and VESA1 export-associated protein, BbVEAP ([33,63]; Fig 2). Up-regulation of SBP2t11 was associated with low virulence of *B*. *bovis*, and its overexpression reduced binding of iRBCs to endothelial cells [63,64]. While the cleavage at PLM and the export of SBP2t11 has been demonstrated [63], it is likely involved in cytoadhesion indirectly, as it is not expressed on the surface of iRBC. It remains unclear whether SBP2t11 affects VESA1 export, ridge formation, or other factors responsible for cytoadhesion. Knockdown of BbVEAP using the *glmS* ribozyme system disrupted the export of VESA1, decreased ridge numbers, and abrogated cytoadhesion of iRBCs [33]. BbVEAP may function as a chaperone for the export of VESA1 as an integral protein and ridge-forming proteins; however, immunoprecipitation of BbVEAP did not confirm a direct interaction with VESA1 [33]. Given that BbVEAP knockdown did not affect SBP4 export, BbVEAP expression is necessary for the export and correct localization of a subset of proteins including VESA1 and ridge-forming proteins. Additionally, it was shown that BbVEAP is indispensable for parasite development in the RBC, making it the only known essential exportome protein [33,65]. The existence of VEAP among piroplasma parasites indicates a piroplasma-specific conserved function. The essentiality of VEAP could be due to its role in the export of other essential proteins such as channels or transporters, a hypothesis that needs future confirmation.

**Fig 4:**
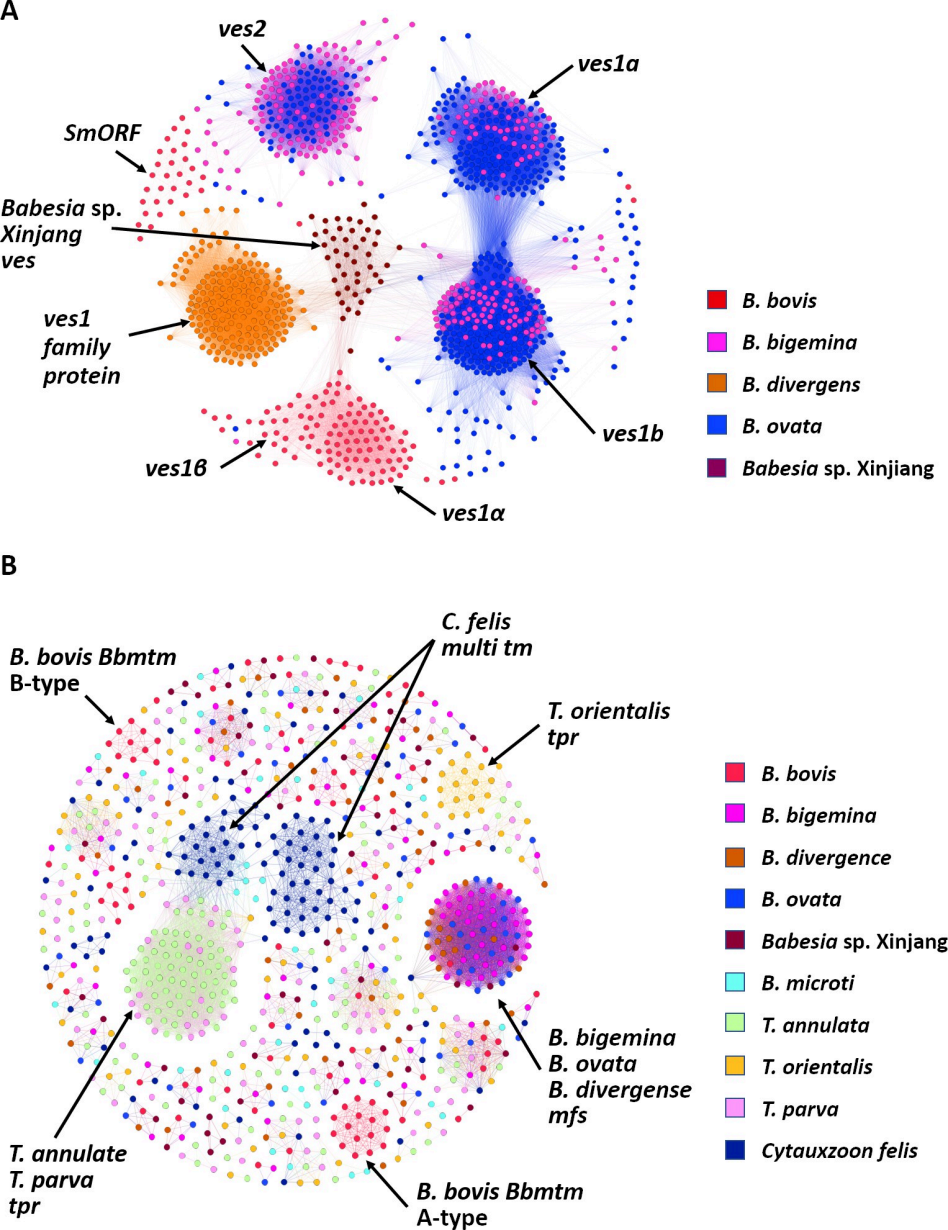
Homology clustering based on sequence similarities of *ves* and multi-transmembrane protein encoding genes. **(**A) *ves* and *smorf* genes sequence were extracted from piroplasmaDB (https://piroplasmadb.org/piro/app), and a sequence similarity network was visualized by Gephi. (B) The figure was reproduced from Hakimi and colleagues [33]. The genes encoding proteins with more than 8 TM domains were extracted from piroplasmaDB and clustered. SmORF, Small Open Reading Frame; TM, transmembrane.

Although *ves1α* and *ves1β* genes are unique for *B*. *bovis*, *ves*-like genes are found in homologous genomic regions in all *Babesia* species for which genome sequence is available (Fig 4A). *Ves1a* and *ves1b* genes are found in *B*. *bigemina*, *B*. *divergens*, and *B*. *ovata* and encode proteins that contain a single transmembrane domain and a short cytoplasmic region at C-terminus similar to the products of *ves1α* and *ves1β* genes of *B*. *bovis* [66,67]. A shorter *ves* gene group called *ves2* encode proteins lacking the C-terminal transmembrane domain and the cytoplasmic region and were found based on their homology to the 5′ end of *ves1* in *B*. *bigemina*, *B*. *divergens*, and *B*. *ovata* [66,67]. The expanded *ves2* genes of *B*. *ovata* cluster together with *B*. *bigemina ves2* based upon high sequence identity (Fig 4A). Given the fact that *ves2* exists in the homologous positions of *smorf*, it was suggested that *ves2* is analogous to *smorf* in these parasites [66]; however, the function of both gene families is unknown. VESA1 from *B*. *bovis* was experimentally confirmed to be expressed on the iRBCs surface [28], and VESA1 from *B*. *orientalis* was shown to be exported to iRBC [68]. While in *B*. *bovis* VESA1 is responsible for cytoadhesion and antigenic variation [69], cytoadhesion has not been documented for other *Babesia* spp., despite the presence of expanded *ves* gene families in their genomes. It is likely that VESA from other *Babesia* species are also surface proteins responsible for antigenic variation [66]. Future experiments are needed to characterize the functions of VESA in *Babesia* spp. in the mammalian host and tick vector, as the expression of some *ves1* genes were up-regulated in the kinetes, the invasive stage of parasites in the tick hemolymph, of *B*. *bovis* [51].

### Nutrient uptake

Mammalian RBCs are terminally differentiated and anucleate and lack cellular machinery and metabolism. *Babesia* parasites have a fast metabolism and require nucleic acids and proteins, and the relatively short doubling time (10 to 12 h for *B*. *bovis*) and DNA replication (2.9 h for *B*. *bovis*) [65] necessitate uptake of metabolic precursors from the host plasma, such as sugars, purines, and amino acids. For *P*. *falciparum* iRBCs, studies using osmotic lysis, patch-clamp methods, and radioactive labeled substrates have documented increases in permeabilities to various nutrients, called the new permeability pathway or plasmodial surface anion channel (PSAC) [70,71]. Similarly, it has been reported that *Babesia* parasites increase RBC permeability to several solutes to facilitate the uptake of essential nutrients for their growth. Sorbitol uptake by RBCs infected with *B*. *divergens* and *B*. *bovis* was shown through osmotic lysis and transmittance assay and was used for the enrichment of iRBCs [33,72]. The uptake of glucose, nucleosides, and several amino acids was shown for several *Babesia* spp. [72–77]. While PSAC is an anion channel, the temperature dependence and relatively slower uptake of solutes by *Babesia* in comparison to malaria parasite suggest a carrier-type nature of the putative channels [72,78].

PSAC activity is determined by the CLAG3 protein [79,80], which is released during merozoite invasion. Orthologs of this protein appear to be absent in *Babesia* parasites. Recently, a protein encoded by a multicopy gene family (Fig 4B) with a multi-transmembrane structure (mtm) was identified in *B*. *bovis* using proteomics of the iRBC membrane ([33]; Fig 2). The expression level of *mtm* correlated with blasticidin S (BS) resistance, suggesting a role in BS uptake [33]. While several *mtms* are expressed simultaneously, only the expression of a single *mtm* was linked with BS resistance [33]. This may indicate that each mtm is involved in the transport of a specific substrate or they have multiple functions. BS resistance in *P*. *falciparum* results in a strong impairment of PSAC activity, as evidenced by a significant reduction of sorbitol uptake and being refractory to synchronization using sorbitol [81], while in *B*. *bovis*, sorbitol uptake and RBC lysis were slightly delayed. This suggests that while PSAC activity is determined by a single CLAG protein, the increase in RBC permeability by *B*. *bovis* is determined by a combination of several mtms. The observation of the simultaneous expression of a subset of mtms supports this hypothesis.

Although *mtm* genes only exist in *B*. *bovis* and *Babesia* sp. *Xinjiang*, expanded gene families that encode proteins with multi-TM structures are found in all piroplasms (Fig 4B; [33]). *B*. *bigemina*, *B*. *ovata*, and *B*. *divergens* have an expanded major facilitator superfamily *mfs*, while *tpr* (*Theilaria parva* repeat), which was initially discovered in *T*. *parva*, is expanded in *Theileria* spp. (Figs 2 and 4B; [82,83]). Whether *mtm* or other expanded multi-TM gene families are responsible for host cell permeability by piroplasms remains to be determined. *Babesia cabali*, the causative agent of equine piroplasmosis, produces tubular structures in the cytoplasm of infected RBCs, which appear to connect the parasite to the host serum [84]. It is not known if these tubular structures are responsible for nutrient uptake as a direct “duct”, and the genes responsible for the generation of these microtubules have not been determined.

## Conclusions

**Fig 5:**
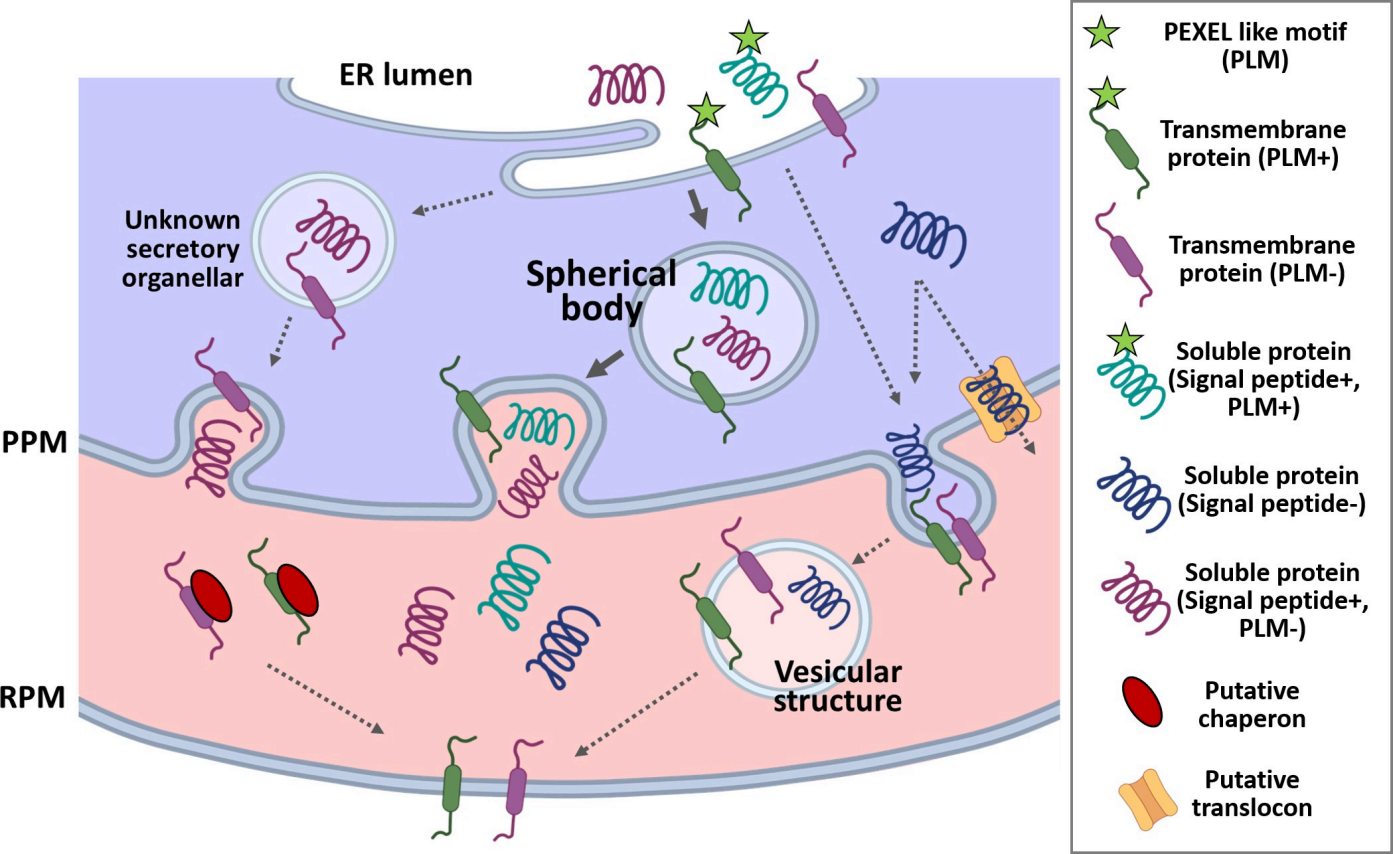
Schematic of the protein export pathway in *Babesia* iRBC. The appointed soluble proteins for the export are being recruited into ER, cleaved by signal peptidase, and likely followed by cleavage of PLM. Transmembrane-containing proteins such as VESA1 or mtm are inserted into the ER membrane. These proteins are loaded into secretory vesicles of the ER-Golgi pathway, which are being transferred to spherical bodies (in the case of spherical body proteins: SBP1, SBP2, SBP3, SBP4, VEAP, and mtm) or directly to parasite plasma membrane (in the case of VESA1). Soluble proteins are released to RBC cytoplasm, while integral protein needs to be extracted from PPM, which may involve protein translocation. Soluble protein could reach the target by diffusion, while membrane proteins are carried out to the target destination through vesicles or in complex with chaperones. It is noted that this scheme is speculative. ER, endoplasmic reticulum; iRBC, infected RBC; PLM, PEXEL-like motif; PPM, parasite plasma membrane; RBC, red blood cell; RPM, RBC plasma membrane; VESA1, variant erythrocyte surface antigen 1.

Exportomes have fundamental roles in apicomplexan parasite survival and virulence, but studies on the *Babesia* exportome have lagged behind. The recent completion of genome databases for multiple *Babesia* species and the development of transfection tools (reviewed in [85]) have allowed us to begin to describe *Babesia* exportomes. Several proteins have been identified from bioinformatic or proteomics approaches and verified by expression of the tagged candidate proteins. However, the proteins responsible for ridge formation, modification of the iRBCs cytoskeleton, and transport to the surface of iRBCs have not been identified. Trafficking of integral proteins such as VESA1 and mtm across the parasite plasma membrane likely require protein refolding, which could be performed by parasite-encoded chaperones (Schematic of the protein export pathway is summarized in Fig 5). While *P*. *falciparum* has an expanded chaperone repertoire containing the domain DNAJ, of which some members are exported to RBCs [86], similar proteins have not been found in *Babesia*. PLM have been identified in hundreds of *Babesia* proteins including all currently known spherical body proteins, which are exportome proteins. Thus, PLM is a powerful tool to predict the *Babesia* exportome. However, some of the experimentally confirmed proteins do not possess a PLM (Table 1), and it is unclear whether PLM in the middle or at C-terminus of the proteins are functional. Further intensive studies using in silico analysis and proteomics will be necessary, and classical approaches such as proteomic analysis of spherical bodies may be also useful. Studies in *Plasmodium* and *Toxoplasma* have shown that dense granule proteins are responsible for host cell modification, and similarly, most of the experimentally confirmed *Babesia* proteins are deposited in spherical bodies and routed to the host RBC. Genetic manipulation tools such as genome editing and inducible gene knockdown/knockout system will be helpful to characterize the functions of proteins identified by in silico screens or proteomics.

Most of the *Babesia* exportome studies have been conducted on *B*. *bovis*, and exportome research on the other *Babesia* spp. have been relatively neglected. Research on *B*. *bovis* is crucial because *B*. *bovis* iRBC have altered morphologies and their sequestration in deep tissues cause pathologies such as cerebral babesiosis. Many of the *Babesia*-predicted exported proteins are encoded by multigene families that are species specific, such as *smorf* and *mtm* that exist in *B*. *bovis*. However, expanded families of *mfs* genes are unique for *B*. *ovata*, *B*. *bigemina*, and *B*. *divergens*, but not found in *B*. *bovis*. Many of the *Babesia* parasites have expanded *ves* genes. TPR-related genes are found in *B*. *bovis*, identified by homology to the *tpr* genes originally described as expanded in *Theileria* spp. Most of the products encoded by these genes are candidate exportome proteins; however, their localization and function have not been studied. Comparative studies on these proteins will give us the information on the evolution and adaptation of each species.

Further identification and functional analysis of the *Babesia* exportome will aid in finding new strategies to control babesiosis. For example, VEAP was suggested to be essential for intraerythrocytic *Babesia* survival and is associated with parasite virulence. In contrast to VESA1, which is highly variable within its multigene family and therefore less amenable to therapeutic designs, VEAP is encoded by a single gene and is conserved among *Babesia* spp. Identification and characterization of such essential and conserved molecules for *Babesia* spp. will reveal basic biology of the parasite and provide candidate targets for vaccination or chemotherapy.
